# A Systematic Review: Do the Use of Machine Learning, Deep Learning, and Artificial Intelligence Improve Patient Outcomes in Acute Myocardial Ischemia Compared to Clinician-Only Approaches?

**DOI:** 10.7759/cureus.43003

**Published:** 2023-08-05

**Authors:** Binay K Panjiyar, Gershon Davydov, Hiba Nashat, Sally Ghali, Shadin Afifi, Vineet Suryadevara, Yaman Habab, Alana Hutcheson, Ana P Arcia Franchini

**Affiliations:** 1 Internal Medicine, California Institute of Behavioral Neurosciences & Psychology, Fairfield, USA; 2 Psychiatry and Behavioral Sciences, California Institute of Behavioral Neurosciences & Psychology, Fairfield, USA

**Keywords:** acute coronary syndrome, acute myocardial infarc, acute st-elevation myocardial infarction, ai and machine learning, ai in cardiology, atherosclerotic cardiovascular disease, cardiac markers, deep learning artificial intelligence, heart attack, interventional cardiology

## Abstract

Cardiovascular diseases (CVDs) present a significant global health challenge and remain a primary cause of death. Early detection and intervention are crucial for improved outcomes in acute coronary syndrome (ACS), particularly acute myocardial infarction (AMI) cases. Artificial intelligence (AI) can detect heart disease early by analyzing patient information and electrocardiogram (ECG) data, providing invaluable insights into this critical health issue. However, the imbalanced nature of ECG and patient data presents challenges for traditional machine learning (ML) algorithms in performing unbiasedly. Investigators have proposed various data-level and algorithm-level solutions to overcome these challenges. In this study, we used a systematic literature review (SLR) approach to give an overview of the current literature and to highlight the difficulties of utilizing ML, deep learning (DL), and AI algorithms in predicting, diagnosing, and prognosis of heart diseases. We reviewed 181 articles from reputable journals published between 2013 and June 15, 2023, focusing on eight selected papers for in-depth analysis. The analysis considered factors such as heart disease type, algorithms used, applications, and proposed solutions and compared the benefits of algorithms combined with clinicians versus clinicians alone. This systematic review revealed that the current ML-based diagnostic approaches face several open problems and issues when implementing ML, DL, and AI in real-life settings. Although these algorithms show higher sensitivities, specificities, and accuracies in detecting heart disease, we must address the ethical concerns while implementing these models into clinical practice. The transparency of how these algorithms operate remains a challenge. Nevertheless, further exploration and research in ML, DL, and AI are necessary to overcome these challenges and fully harness their potential to improve health outcomes for patients with AMI.

## Introduction and background

Cardiovascular diseases (CVDs) have been the cause of global deaths for decades. In 2021, around 20.5 million people died due to CVDs, accounting for approximately one-third of all global deaths [1]. Ischemic heart (IHD) is the leading cause of early mortality in men in 146 nations and women in 98 countries. The deaths due to cardiovascular disease increased from 12.1 million in 1990 to 20.5 million in 2021, as reported by the World Heart Federation [2]. Heart attacks occur in the United States every 40 s [3]. It is significant to research chronic illnesses that involve recognizing biomarkers and classifying potential risks. Utilizing advanced algorithms such as machine learning (ML), deep learning (DL), and artificial intelligence (AI) can prove to be highly advantageous in achieving these objectives.

A heart attack occurs when blood flow to the heart muscle is interrupted. This can happen if a coronary artery is partially or completely blocked [4]. Acute myocardial infarction (AMI) is classified into two types based on electrocardiogram (ECG) findings: ST-segment elevated myocardial infarction (STEMI) and non-ST-segment elevated myocardial infarction (NSTEMI) [4]. Complete blockage of the coronary artery leads to STEMI. Patients with NSTEMI have a partial blockage and no visible ST-elevation on ECG. STEMI patients have a greater mortality risk than NSTEMI patients [4].

Multiple factors can contribute to AMI, including smoking, hypertension, high BMI, hyperglycemia, an unhealthy diet, and genetic predispositions, leading to dyslipidemia, and the harmful use of substances such as alcohol and drugs, as well as physical inactivity [5]. Post-MI patients can be affected by various factors, including non-communicable diseases, demographics, and environmental factors, which can impact AMI mortality [6,7]. AMI often comes with chest pain, a common symptom often reported [8]. Chest pain can have many causes, so physicians must diagnose life-threatening conditions quickly and accurately. ACS is a group of diagnoses related to coronary ischemia, including unstable angina (UA), NSTEMI, and STEMI [9]. We need to urgently diagnose and treat ACS to improve outcomes and reduce mortality and morbidity caused by ACS [10].

It is important to understand that not all patients who experience chest pain in the emergency room are suffering from acute coronary syndrome (ACS) [11]. Therefore, risk stratification is crucial to evaluating chest pain [12]. Relying solely on a patient's medical history and physical examination can be unreliable [13]. There are different conventional models available to help with risk stratification, such as thrombolysis in myocardial infarction (TIMI) [14], Framingham risk score (FRS) [15], global registry of acute coronary events (GRACE) [16], and history, ECG, age, risk factors, and troponin (HEART) [17]. When choosing models for different features, it is essential to consider specific requirements and complex results. Although decision tools have undergone multiple trials for validation, there is still a possibility of missing some cases of ACS. That is where the emerging field of artificial intelligence (AI) can significantly impact the practice of medicine soon [18,19]. There has been a desire to utilize AI-based methods to assess chest pain for quite some time [20].

AI is concerned with creating computer systems capable of doing activities that typically need human intelligence [21]. In the past decade, research on AI has made significant progress due to advancements in computing power, digital data, and improved algorithms [22]. A subfield of AI called ML uses different techniques to recognize patterns in data and make predictions or decisions automatically [23]. ML models improve performance by continuously comparing their predictions with actual results and adjusting their internal parameters accordingly. Deep learning (DL), a subclass of ML, utilizes interconnected non-linear processing units to create increasingly abstract data representations, enabling it to learn how to model complex tasks [24].

Despite their current limitations in application, advanced ML technologies have achieved remarkable success in solving problems that were once thought to be unsolvable [25]. Although more versatile models are being developed, current narrow ML technologies have the potential to impact various industries, especially healthcare significantly [26]. Using AI techniques, patient outcomes can be predicted, and patients can be categorized based on their clinical and physiological data. Recent advancements have even led to accurate diagnoses of myocardial infarction [27]. The integration of AI techniques into clinical practice still presents a challenge.

ML is a subclass of AI that simulates human learning and problem-solving using data and algorithms, improving its accuracy via learning [28]. Acute Myocardial Infarction (AMI) risk classification has been greatly aided by ML, with popular algorithms such as logistic regression (LR), support vector machines (SVM), K-nearest neighbor (KNN), artificial neural network (ANN), and random forest (RF) [29]. ML falls into three distinct types: supervised (labeled data), unsupervised (unlabeled data), and reinforcement (feedback from rewarding behaviors) [29].

This systematic review evaluates available evidence of the effectiveness of ML in acute myocardial ischemia by answering the question: Can machine learning models surpass the accuracy of doctors or current risk assessment methods?

## Review

Methods

**Figure 1:**
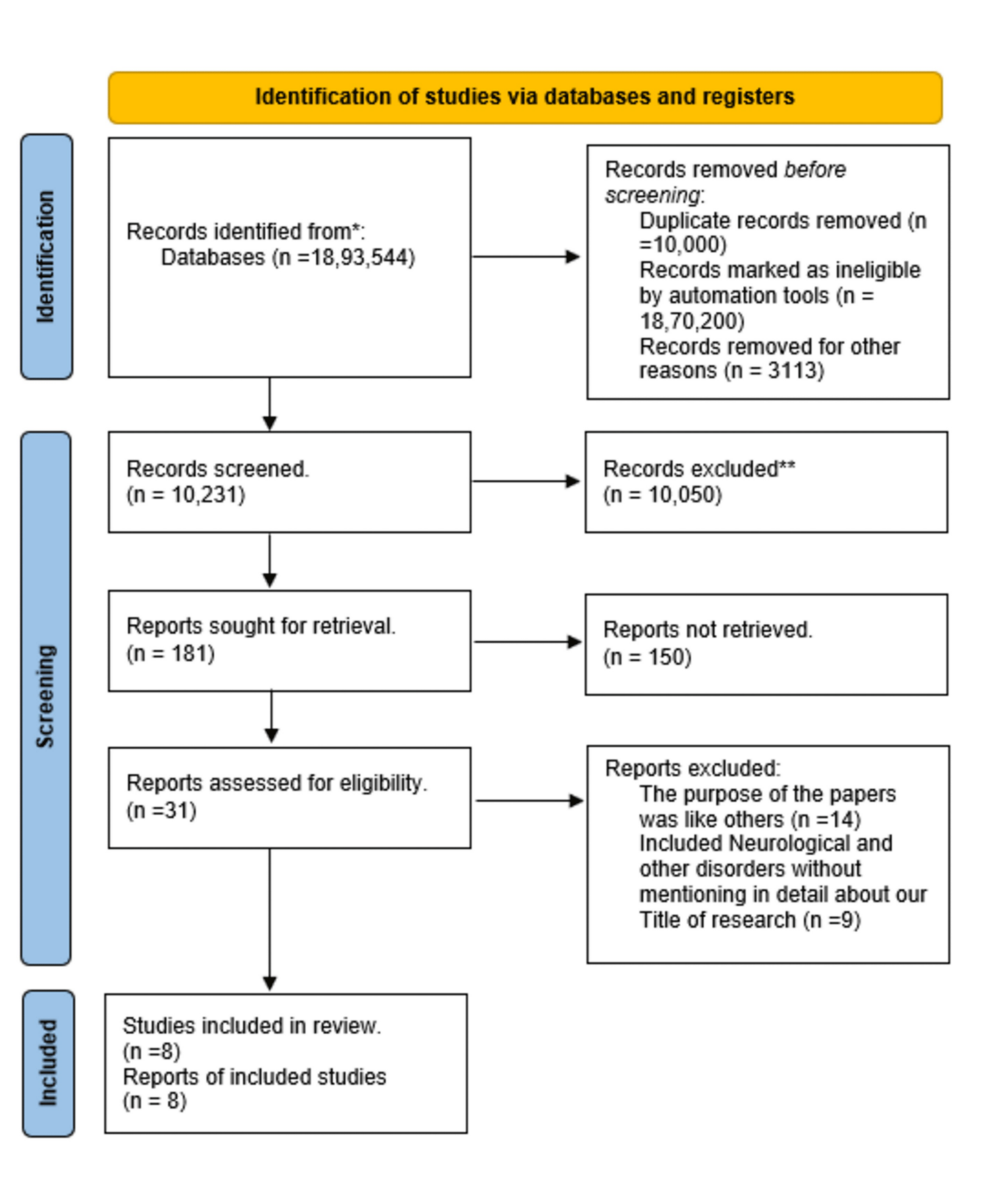
PRISMA flow diagram illustrating the search strategy and study selection process for the systematic review. PRISMA: Preferred Reporting Items for Systematic Reviews and Meta-Analyses

This review focuses on clinical studies concerning the use of ML/DL/AI in cardiovascular medicine. We excluded animal studies and publications that only discussed the methodology of AI/ML/DL without presenting clinical data. The review follows the guidelines for Preferred Reporting Items for Systematic Reviews and Meta-Analyses (PRISMA) [30] for 2020 in Figure 1 and only uses data collected from published papers, eliminating the need for ethical approval.

Systematic Literature Search and Study Selection

We conducted a thorough search for relevant publications by using PubMed, including Medline and Google Scholar. We searched for studies mentioned in review papers, editorials, and commentaries on PubMed. Nevertheless, we continued searching for additional studies that satisfied our inclusion criteria.

We had a list of abstracts that we independently reviewed for inclusion using specific criteria. The criteria included using AI/ML methods, focusing on cardiovascular applications, and a clearly described clinical cohort in the study. We excluded review papers and animal studies. Six reviewers conducted a dual review, and disagreements were resolved through discussion.

Inclusion and Exclusion Criteria

We established specific criteria for including and excluding participants to achieve our study goals. Our Criteria can be summarized in Table 1.

**Table 1:** Showing the criteria adopted during the literature search process.

	Inclusion Criteria	Exclusion Criteria
a)	Human studies	Animal studies
b)	From 2013 to 2023	Only methodological studies explaining programming details
c)	English Text	Non-English texts
d)	Gender: All	
e)	Age: > 13 years of age	Age:<=13 years
f)	Free papers	Papers that needed to be purchased
		Studies involving clinical data other than cardiovascular diseases

Search Strategy

The population, intervention/condition, control/comparison, and outcome (PICO) criteria were utilized to conduct a thorough literature review. The search was conducted on databases such as PUBMED (including Medline) and Google Scholar Libraries, using relevant keywords, such as acute myocardial ischemia, ML, DL, and AI. The medical subject heading (MeSH) approach for PubMed (including Medline) and Google Scholar, as detailed in Table 2, was employed to develop a comprehensive search strategy.

**Table 2:** Showing the search strategy, search engines used, and the number of results displayed. ML: Machine learning; DL: Deep learning; AI: Artificial intelligence

	Database	Search Strategy	Search Results
A)	PubMed	Ischemic Heart Disease and Machine Learning	777
		Ischemic Heart Disease and Machine Learning and Deep Learning and Artificial Intelligence	137
		Diagnosis or Treatment of Ischemic Heart Disease and Machine Learning or Deep Learning or Artificial Intelligence	18,75,330
B)	Google Scholar	ML, DL, AI, and Ischemic Heart Disease	17,300

Quality Appraisal

To ensure the reliability of our chosen papers, we utilized various quality assessment tools. We employed the PRISMA checklist and Cochrane bias tool assessment for randomized clinical trials for systematic reviews and meta-analyses. Non-randomized clinical trials were evaluated using the Newcastle-Ottawa tool scale. We assessed the quality of qualitative studies, as shown in Table 3, using the critical appraisal skills program (CASP) checklist. To avoid any confusion in the classification, we utilized the scale for the assessment of narrative review articles (SANRA) to evaluate the article's quality.

**Table 3:** Showing quality appraisal tools used. PRISMA: Preferred reporting items for systematic reviews and meta-analyses; SANRA: Scale for the assessment of non-systematic review articles

Quality Appraisal Tools Used	Type of Studies
Cochrane Bias Tool Assessment	Randomized Control Trials
Newcastle-Ottawa Tool	Non-RCT and Observational Studies
PRISMA Checklist	Systematic Reviews
SANRA Checklist	Any Other Without Clear Method Section

Results

A comprehensive literature search across multiple databases yielded a total of 1,893,544 records. Following PRISMA guidelines, a systematic multi-stage screening process was carried out. Before screening began, 10,000 duplicate records were removed, 1,870,200 records were automatically excluded as ineligible, and 3,113 were removed for other reasons. This resulted in 10,231 records eligible for title and abstract screening, from which 10,050 were excluded for not meeting the inclusion criteria. Of the remaining 181 records, full-text articles were sought, but 150 could not be retrieved. The remaining 31 articles underwent full-text eligibility assessment. Fourteen studies were excluded due to overlapping content with already included studies, while nine were excluded for lacking sufficient detail on the study’s target topic; these were more broadly focused on neurological and other unrelated disorders. Ultimately, eight studies met all inclusion criteria and were included in the final qualitative synthesis. Table 4 provides a detailed description of each.

**Table 4:** Summary of the results of the selected papers. AUC: Area under curve; CAD: Coronary artery disease; DL: Deep learning; AI: Artificial intelligence; ACS: Acute coronary syndrome; STEMI: St-elevated myocardial infarction; NSTEMI: Non st-elevated myocardial infarction

Author/Year	Country	Study Design	Database Used	Conclusion
Ahsan et al./2022 [31]	USA	Systematic Literature Review	Scopus	They aim to create machine learning systems for accurate heart disease diagnosis using real-time patient data.
Al Hinai et al./2021 [32]	Canada	Systematic Review	PubMed and Medline	DL models can accurately analyze resting ECG signals to detect heart disease.
Alizadehsani et al./2019 [33]	USA, Canada, Australia	Database Highlights	Google Scholar	A new CAD database was created from 126 papers and 68 datasets on CAD diagnosis from 1992 to 2018.
Friedrich et al./2021 [34]	Germany	Systematic Review	PubMed and Embase	Efficient AI/ML methods are underutilized in clinical practice. Medical challenges can advance research and promote their usage.
Krittanawong et al./2020 [35]	USA	Meta-Analysis	Medline, Embase, and Scopus	The study analyzed 55 studies with over 3 million individuals to assess the impact of machine learning on cardiovascular diseases. Specifically, 45 of these studies focused on coronary artery disease (CAD) and showed that custom-built algorithms had a higher AUC and sensitivity than boosting algorithms.
Nagendran et al./2020 [36]	UK	Systematic Review	They searched through databases such as Medline, Embase, Cochrane Central Register of Controlled Trials, and the World Health Organization trial registry.	A total of 61 out of 81 studies found that AI performed as well as or better than clinicians, but only 38% of them suggested follow-up studies.
Panteris et al./2022 [37]	Greece	Randomized Clinical Trial	Out of 960 patients in a clinical trial, 533 had ACS, 170 had NSTEMI, 222 had STEMI, 141 had unstable angina, and 681 had obstructive CAD.	A machine learning model accurately predicts obstructive CAD likelihood by combining metabolic and clinical features. XGBoost algorithm achieves an impressive AUC of 0.725.
Stewart et al./2021 [38]	Australia	Systematic Review	They searched databases and reviewed 3361 articles, ultimately including 23 in the analysis.	More research is needed before artificial neural networks (ANN) can be widely used in clinical settings to detect heart attacks and forecast cardiovascular incidents.

Discussions

Early detection is critical in improving the survival and outcomes of coronary artery disease (CAD). Researchers have created models that predict which patients are at a higher risk using techniques such as ML and data mining (DM) [39]. These advanced techniques help quickly analyze large amounts of medical data and reveal hidden structures, allowing for a more efficient and semi-automated approach to finding patterns in data [40]. Several commonly used prediction models, such as the Framingham risk score [41], sex-specific and race-specific pooled cohort equations (PCE) model [42], systematic coronary risk evaluation (SCORE) [43], and QRESEARCH cardiovascular risk algorithm (QRISK) [44], are utilized to identify various predictive factors. However, these models have limitations, such as differences among validation cohorts and the possibility of overestimating the risk of CVD. Furthermore, these models often need to pay more attention to important variables, making less accurate predictions.

To overcome limitations in specific populations like those with rheumatoid arthritis, more advanced prediction tools are necessary [45]. The growing computational power has led to a surge in interest in risk prediction based on ML. However, clinicians may require a deeper understanding of this methodology. Accurate risk prediction is crucial, and further research is needed to improve the accuracy of CVD burden prediction. Organizing research and findings on CAD diagnosis has been challenging due to the need for publicly available comprehensive benchmarks. To address this issue, creating a database that collects all related studies and their information could be a solution. Thus, the created database would inform researchers about the latest developments and methods proposed in the field. A group of researchers, Alizadehsani et al. [33], has made progress by creating a thorough dataset for ML/DM and detecting heart disease. This database holds critical data and results from ML algorithms for understanding CAD disease.

According to Alizadehsani et al.'s study, utilizing an SVM can effectively differentiate individuals with CAD from those who are healthy, with a 95% level of accuracy [33]. Research emphasizes that age, hypertension, chest pain, regional wall motion abnormality, and ejection fraction are crucial to diagnosing CAD. This information can benefit biologists, healthcare researchers, computer scientists, and physicians specializing in CAD. In addition, combined analyses suggest that ML algorithms are typically precise when forecasting prevalent cardiovascular illness, achieving an AUC value of 0.8-0.9. Subgroup analyses indicate that ML algorithms accurately predict CAD and stroke [35]. A study by Liu et al. [46] compared the diagnostic performance of DL models and clinicians using medical imaging. The results indicated that DL algorithms show potential. However, specific methodological barriers must be addressed to achieve clinician-level accuracy.

Metabolic profiling data on cardiovascular diseases have increased in the past decade. Though some risk scores based on metabolites have been developed, it has yet to translate into clinical benefits due to the complexity of metabolomics data [47,48]. ML in metabolomics studies has become more widespread due to its capability to handle complex and diverse data and non-linear data representation [47,48]. ML is a specific category of AI proficiently performing these tasks. Despite its infancy in cardiovascular medicine, ML has already been utilized to identify unknown CAD risk factors, automate imaging interpretation, facilitate precision medicine, and enhance clinical decision-making [35,49,50]. ML could enable clinicians to provide better diagnosis, risk stratification, and manage CAD patients by improving CAD prediction accuracy. At present, no ML method focuses on clinical applications and analyzes metabolic markers to forecast obstructive CAD in patients who are undergoing invasive coronary angiography (ICA) [51,52].

ML approaches aim to interpret how risk factors affect outcomes [53]. A recent meta-analysis of 45 cohorts showed that ML algorithms outperformed traditional regression analyses in predicting CAD. Using boosting algorithms resulted in a combined AUC score of 0.88, with a sensitivity of 0.86, and a specificity of 0.70. Studies have shown that ML models outperform conventional risk classification models by identifying hidden patterns and including various data types [35]. For example, ML models have improved the risk classification of relevant subpopulations, including individuals with a history of diabetes, and have shown better performance in mortality predictions for STEMI than the TIMI model [54]. Therefore, ML could be better than conventional CVD risk classification methods [54].

Research has demonstrated that ML algorithms, explicitly boosting algorithms and SVMs, are effective in cardiovascular medicine. To ensure accurate data interpretation, it is crucial to select appropriate algorithms, compare their performance with that of human experts, and report all evaluation metrics. Further studies should be conducted to compare ML algorithms with traditional risk models. Once validated, these algorithms can be incorporated into electronic health record systems to enhance clinical practice [35].

Logistic regression was more effective than ML techniques by Tsien et al. [55] in 1998 and Harrison et al. [56] in 2005. They proposed that clinical practice models could be developed with only a few data points. The "MI3 clinical support tool" created by Than et al. used ML to diagnose type 1 myocardial infarction with high accuracy (AUC of 0.963) [57]. A recent study has found that using troponin tests simultaneously can effectively identify low or high-risk chest pain. In diagnosing type 1 myocardial infarction, this approach is more precise than the pathway recommended by the European Society of Cardiology. The test has a 99.7% negative predictive value for low-risk patients and 97.8% sensitivity. The test has a 71.8% positive predictive value and 96.7% specificity for high-risk patients [57].

In four different studies, the use of ML was compared to that of a physician in diagnosing AMI cases. One study by Chazaro et al. [58] found that the Emergency Department (ED) physician had higher sensitivity (87%) compared to the ANN (85%) but lower specificity (78% vs. 91%). In contrast, the ANN performed better than the physician in all areas in the remaining three studies [59-60]. In the 1990s, Baxt et al. [60] have been found through their research that non-linear ANNs exhibit greater accuracy than both human doctors and other computer-based methods. However, limited study has compared the effectiveness of ML and physicians [61]. In the ED, ML techniques are commonly utilized to diagnose and predict outcomes for patients with chest pain. These highly effective techniques lead to improved patient care and greater efficiency in healthcare services. Owing to recent technological advancements, larger datasets and more accessible ML models can be trained in seconds [61].

No recent study compares the accuracy of ML and physicians in diagnosing undifferentiated chest pain in EDs. In recent studies, ML has been compared to the current risk stratification tools such as the TIMI and history, ECGs, age, risk factors, troponins (HEART) scores. While the HEART score is often utilized in clinical settings, recent evidence indicates that it may not be superior to clinical judgment in specific clinical scenarios [62]. As ML tools become more commonly used, assessing their effectiveness compared to physicians' consistently is crucial. It is essential to conduct more randomized clinical trials to confirm the accuracy of ML algorithms in identifying the risk level of chest pain. These studies should compare the effectiveness of the algorithms to that of physicians and current risk-scoring tools. Additionally, no studies have assessed the impact of ML algorithms on patient outcomes after implementation in clinical practice. It is crucial to evaluate how these tools affect clinical decision-making. It should be noted that implementing ML algorithms can either decrease or increase bias. Hence, it is critical to establish suitable frameworks for algorithm stewardship to ensure their effective implementation in the future [62]. Adding extra data such as physician notes, ECGs, and X-rays can improve the accuracy of ML models. In patients with acute coronary artery occlusion, immediate reperfusion therapy can be beneficial. However, the current STEMI/NSTEMI paradigm does not identify all patients based on ST elevation on ECGs [63]. While some studies have used angiogram results, none have tried to identify all patients with acute coronary artery occlusion. In upcoming studies, ML could be utilized to identify these patients who do not meet the current STEMI criteria [63].

In the healthcare industry, utilizing ML prediction models, as noted by Zhang et al. [63], can raise ethical and legal concerns. For instance, technology manufacturers and emergency physicians may be liable for malpractice. Moreover, there is a valid concern that algorithmic outputs, which humans may not comprehend [33], may influence essential decisions. Therefore, the legal framework should be updated to address ML-related medical malpractice [64].

Although there have been positive outcomes in using ML algorithms in clinical practice for the past 30 years, the integration process is still challenging. One significant barrier to this is the differences among healthcare systems. Zhang et al. successfully implemented their model, although they acknowledge that it may only be effective in certain hospitals [63]. Their proposed solution is to retrain and test the model in other medical facilities. Than et al. developed an initial version of an application that showcases the potential of utilizing a centralized ML algorithm in an environment with limited resources. The app provides diagnostic metrics to physicians and graphical results to patients via a phone application [57]. Establishing oversight and stewardship frameworks for ML algorithms is crucial for safe, effective, and fair use on diverse patient populations. Continued monitoring of health systems is also necessary for success [65].

Limitations

Our literature review has limitations. We limited our analysis to English articles published within the last 10 years, specifically targeting those at least 13 years old. We also only used free articles, and our study was limited to English papers on ML, DL, and AI for diagnosis and prognosis. More research is needed for specific conclusions.

## Conclusions

Our research highlights the appropriate use of recent DL models to effectively prevent, risk-stratify, and manage CAD patients to optimize their prognosis. These technological advancements aim to alleviate the burden on clinicians and support them. Although challenges still exist, encouraging results have been obtained from prospective and retrospective clinical trials. Some of the researchers have successfully developed an initial version of an application that showcases the potential of utilizing a centralized ML algorithm in an environment with limited resources. These algorithms can guide timely interventions, ultimately saving lives and resources for patients with acute ischemic attacks. However, large-scale RCTs are necessary to establish these algorithmic models' diagnostic and prognostic benefits, and caution should be exercised when using them for preventive purposes.
