# Correction: A Systematic Review: Do the Use of Machine Learning, Deep Learning, and Artificial Intelligence Improve Patient Outcomes in Acute Myocardial Ischemia Compared to Clinician-Only Approaches?

**DOI:** 10.7759/cureus.c307

**Published:** 2025-09-23

**Authors:** Binay K Panjiyar, Gershon Davydov, Hiba Nashat, Sally Ghali, Shadin Afifi, Vineet Suryadevara, Yaman Habab, Alana Hutcheson, Ana P Arcia Franchini

**Affiliations:** 1 Internal Medicine, California Institute of Behavioral Neurosciences & Psychology, Fairfield, USA; 2 Psychiatry and Behavioral Sciences, California Institute of Behavioral Neurosciences & Psychology, Fairfield, USA

This article has been corrected due to errors in the first paragraph of the Results section describing the search strategy. This paragraph has been revised as follows:

Old (incorrect): After searching through three selected databases, PubMed, Medline, and Google Scholar, we extracted 1,893,544 articles. We then carefully reviewed each paper and applied specific criteria, which led to excluding 18,20,323 articles. From the remaining 20,381 papers, we chose not to utilize 20,200 of them due to duplicates or unsatisfactory titles and abstracts. We closely examined the remaining 181 papers and excluded 173 more as their content did not meet our inclusion criteria. Finally, we conducted a thorough quality check on the remaining eight papers, which all met our criteria. These eight articles are included in our final systematic review. Table 4 provides a detailed description of each.

New (correct): A comprehensive literature search across multiple databases yielded a total of 1,893,544 records. Following PRISMA guidelines, a systematic multi-stage screening process was carried out. Before screening began, 10,000 duplicate records were removed, 1,870,200 records were automatically excluded as ineligible, and 3,113 were removed for other reasons. This resulted in 10,231 records eligible for title and abstract screening, from which 10,050 were excluded for not meeting the inclusion criteria. Of the remaining 181 records, full-text articles were sought, but 150 could not be retrieved. The remaining 31 articles underwent full-text eligibility assessment. Fourteen studies were excluded due to overlapping content with already included studies, while nine were excluded for lacking sufficient detail on the study’s target topic; these were more broadly focused on neurological and other unrelated disorders. Ultimately, eight studies met all inclusion criteria and were included in the final qualitative synthesis. Table 4 provides a detailed description of each.

